# Comparison of Body Composition Assessed by Dual-Energy X-Ray Absorptiometry and BMI in Current and Former U.S. Navy Service Members

**DOI:** 10.1371/journal.pone.0132157

**Published:** 2015-07-21

**Authors:** Heath G. Gasier, Linda M. Hughes, Colin R. Young, Annely M. Richardson

**Affiliations:** 1 Center for Hyperbaric Medicine and Environmental Physiology, Duke University Medical Center, Durham, North Carolina, United States of America; 2 Naval Submarine Medical Research Laboratory, Groton, Connecticut, United States of America; 3 Department of Pediatrics, Baystate Children’s Hospital, Springfield, Massachusetts, United States of America; University of Leipzig, GERMANY

## Abstract

**Background:**

Little is known of the diagnostic accuracy of BMI in classifying obesity in active duty military personnel and those that previously served. Thus, the primary objectives were to determine the relationship between lean and fat mass, and body fat percentage (BF%) with BMI, and assess the agreement between BMI and BF% in defining obesity.

**Methods:**

Body composition was measured by dual-energy X-ray absorptiometry in 462 males (20–91 years old) who currently or previously served in the U.S. Navy. A BMI of ≥ 30 kg/m^2^ and a BF% ≥ 25% were used for obesity classification.

**Results:**

The mean BMI (± SD) and BF% were 28.8 ± 4.1 and 28.9 ± 6.6%, respectively, with BF% increasing with age. Lean mass, fat mass, and BF% were significantly correlated with BMI for all age groups. The exact agreement of obesity defined by BMI and BF% was fair (61%), however, 38% were misclassified by a BMI cut-off of 30 when obesity was defined by BF%.

**Conclusions:**

From this data we determined that there is a good correlation between body composition and BMI, and fair agreement between BMI and BF% in classifying obesity in a group of current and former U.S. Navy service members. However, as observed in the general population, a significant proportion of individuals with excess fat are misclassified by BMI cutoffs.

## Introduction

Although a simplistic description, the human body is comprised of fat and fat-free (water, protein and mineral) compartments [1]. The amount that each contributes to total mass can vary with age, gender and race/ethnicity [2, 3]; however, a body fat percentage (BF%) between 12–20% in males and 20–30% in females would historically be viewed as typical [1, 4]. Unfortunately, there is currently an obesity epidemic in the U.S. with 68.2% of adults being either overweight (33.6%) or obese (34.6%) [5]. The rise in obesity is similarly being observed within the U.S. military, i.e., 47.6% classified as overweight and 12.9% obese [6]. These data may suggest that a body composition that has generally been considered “normal” may now actually be uncommon, a scenario that is troubling considering the consequences associated with obesity, i.e., hypertension, dyslipidemia, cardiovascular disease, type 2 diabetes, sleep apnea and cancer [7, 8].

Overweight indicates weighing more than “normal” with the excess weight assumed to be fat mass, whereas obesity is an abnormal accumulation of fat that may impair one’s health [8, 9]. A common indicator that is used to assess the magnitude of body fatness, thus whether someone is overweight or obese, is the body mass index (BMI; body weight in kg divided by height in m^2^) [8, 9]. Although BMI does not directly provide information regarding the quantity of fat and fat-free mass, there is a good relationship between BMI and measured BF% by dual energy x-ray absorptiometry (DXA), Pearson’s *r* > 0.7 [10], and BMI and measured BF% and lean mass by bioelectrical impedance, Pearson’s *r* ~ 0.7 [11]. However, misclassification can occur when using BMI [11, 12], such that one could truly be obese (considered to be a measured BF% > 25% [4]) or possess significantly more fat-free mass. The consequence of misclassification from a global public health perspective is an imprecise understanding of the magnitude of the obesity epidemic.

Several investigations have characterized the prevalence of overweight and obesity in active duty military service members [6, 13, 14] and veterans [15, 16]. The estimates are based on BMI, thus it remains unknown whether members of the armed services and those that previously served are being misclassified as obese when in fact they are not. Therefore, we determined body composition (fat and fat-free mass) by DXA in a group of active duty and former U.S. Navy servicemembers. The primary objectives of this research were to determine the relationship between lean mass, fat mass and BF% with BMI, and assess the agreement in classifying obesity by BMI and BF%. To our knowledge, this is the first investigation that has compared BMI to BF% measured by DXA in classifying obesity in a group of current and former military personnel.

## Materials and Methods

### Subjects

Subjects were males (n = 462) between the ages of 20–91 and currently serving, or previously had served, in the U.S. Navy. As previously described [17], subjects were submariners by occupation and were residing in New England, U.S., at the time of recruitment (2011–2012). This investigation was approved by the Naval Submarine Medical Research Laboratory Institutional Review Board (protocol NSMRL.2011.0001) in compliance with all applicable Federal regulations governing the protection of human subjects. Upon description of study objectives, subjects provided written informed consent to participate in this cross-sectional study.

### Health Related Behaviors

Subjects completed a health screening questionnaire to obtain information regarding past medical history, including smoking status. The activity frequency questionnaire (The University of Arizona Activity Frequency Questionnaire-AAFQ, Tucson, AZ) was administered to assess whether the subjects were obtaining the recommend amount of moderate physical activity for Americans (150 minutes/week or ~20 min/day) [18]. The AAFQ groups physical activity by leisure, recreational, household, other, and accounts for sleep and occupational work [19]. The food frequency questionnaire (General Nutrition Assessment Food Frequency Questionnaire Fred Hutchinson Cancer Research Center, Seattle, WA), which is commonly used for diet assessment [20], was used to determine whether the subjects were meeting the dietary guidelines for Americans [21]. Specifically, the mean daily total fat, saturated fat, cholesterol, sodium, fiber, and servings of fruits and vegetables were calculated by the Nutrition Assessment Shared Resources (NASR; Fred Hutchinson Cancer Center) that uses Nutrition Data Systems for Research software, a US Department of Agriculture Nutrient Database).

### Body Composition Assessment

Standing height (without shoes) and body weight (T-shirt and shorts) were measured with a stadiometer and clinical scale to the nearest 0.1 cm and 0.1 kg, respectively. BMI was calculated as weight (kg) divided by height squared (m^2^), and used to classify subjects as underweight (< 18.5), normal-weight (18.5–24.9), overweight (25–29.9), or obese (> 30) [9]. Upon removal of all jewelry, body composition (fat and fat-free (lean and bone mineral content) mass) was measured with a GE Lunar iDXA fan beam densitometer (GE Healthcare, Madison, WI) using enCORE Version 13.60 software (2011). BF% was calculated from fat mass divided by total mass x 100 for total body, android, and gynoid regions. Currently, there are no BF% categories that define a healthy storage of adipose tissue *vs*. excessive. A BF% ≥ 25 has been suggested to define obesity when objective measurements are employed [4, 11, 12, 22, 23], thus this cutoff was adopted for defining measured obesity in the present investigation. Exams that contained artifacts from prostheses (6 subjects had either a unilateral or bilateral hip replacement) were removed from the analysis, thus body composition data was available on 456 volunteers. Each day scans were performed, a quality assurance test was conducted using a calibration phantom consisting of tissue-equivalent material. Upon conducting repeated measurements on a sample of males (*n* = 12), the coefficient of variation for lean and fat mass and BF% was determined to be 0.6%, 1.6% and 1.8%, respectively.

### Statistical Analysis

Statistical analyses were conducted using SPSS version 19.0 (IBM Corp., Armonk, NY). Categorical demographics (duty status, age and race-ethnicity) and reported health characteristics (smoking status, hypertension, hyperlipidemia and Type 2 diabetes mellitus) are presented by frequencies and percentages. Anthropometrics are reported as mean ± SD and analysis of variance (ANOVA) tests determined if anthropometric means differed by age group. The mean BF% was also calculated by BMI category (i.e., underweight, normal weight, overweight, and obese). Pearson correlation coefficients were calculated to measure the associations between lean mass, fat mass, BF% and BMI. The differences between paired correlations with a variable in common were tested using the Williams t-test method with the Bonferroni adjustment applied to control for type I error’s [24]. BMI and BF% measurements were both dichotomized (not obese/obese) and then cross-classified. Cohen’s kappa (Ck) coefficients were then calculated to assess the extent of agreement between the two measures in determining the prevalence of obesity. Treating measured BF% as the reference method, % of exact agreement also represents the proportion of subjects that were correctly classified as obese or not obese based on their BMI. Finally, the percentage of the subjects that obtained the recommended amount of daily physical activity [18], and were compliant with dietary recommendations for Americans [21] were calculated. Statistical significance was set at *P* < 0.05.

## Results

Demographic and health characteristics of the study participants are displayed in **Table 1**.

**Table 1:** Subject Characteristics. For dietary intake, *n* = 461 and for all others, *n* = 462.

	*n*	Percent
Duty status		
Active duty	340	73.6
Non-active duty	122	26.4
Age group		
20–39 y	297	64.3
40–59 y	90	19.5
≥ 60 y	75	16.2
Race-ethnicity		
Non-Hispanic white	418	90.5
Non-Hispanic black	15	3.2
Hispanic	19	4.1
Other	10	2.2
Obesity related conditions		
Hypertension	63	13.7
Hyperlipidemia	38	8.2
Type II diabetes mellitus	19	4.1
Health behavior		
Current smoker	89	19.3
Physical activity (> 20 min/day)^1^	356	77.1
Dietary intake^2^		
Total fat (< 35% total kcal)	287	62.3
Saturated fat (< 10% total kcal)	127	27.5
Cholesterol (< 300 mg)	246	53.4
Sodium (< 2300 mg)	83	18.0
Fiber^3^	76	16.5
Fruits and vegetables (≥ 5 servings)	140	30.3

^1^Physical Activity Guidelines Advisory Committee Report, 2008 [18].

^2^Dietary Guidelines for Americans, 2010 [21].

^3^Fiber recommendations by age: 34 g (19–30 y), 31 g (31–50 y), and 28 g (≥ 50 y).

The majority of the subjects were active duty submariners (74%) between the ages of 20–39 years old (64%), and were non-Hispanic whites (> 90%). Of the health risk behaviors, most of the subjects reported that they were non-smokers and were obtaining the recommended amount of moderate intensity physical activity. Hypertension was the most commonly reported obesity related condition (14%), followed by hyperlipidemia (8%) and type II diabetes mellitus (4%). While nearly 2/3 of the subjects reported consuming < 35% of their total daily energy from fat, substantially less met the dietary guidelines for saturated fat, cholesterol, sodium, fiber, and fruits and vegetable consumption.

Mean comparisons of anthropometric measures by age group are depicted in **Table 2**. There were significant differences between age groups for all anthropometric measures (*P* < 0.001). BMI, fat mass, android BF%, and the android/gynoid fat ratio were significantly greater in both 40–59 and ≥ 60 year old groups compared to the 20–39 year old group (*P* < 0.01), while the overall mean BF% increased with age (*P* < 0.01). Fat-free and lean mass were significantly greater in the 40–59 year old group compared to 20–39 and ≥ 60 year old groups (*P* < 0.01).

**Table 2:** Mean comparisons of height, body mass and body composition by age group. Values are mean ± SD. For height, weight and BMI, *n* = 297 (20–39 y), 90 (40–59 y) and 75 (≥ 60 y), and for fat-free mass (FFM), lean mass, fat mass, BF% measures, and android/gynoid ratio, *n* = 297 (20–39 y), 88 (40–59 y) and 71 (≥ 60 y).

Parameter	20–39 y	40–59 y	≥ 60 y	Total
Height (cm)	177.5 ± 6.6^a^	178.0 ± 6.8^a^	174.3 ± 6.8^b^	177.1 ± 6.8
Weight (kg)	88.3 ± 13.5^a^	96.9 ± 18.3^b^	91.1 ± 13.3^ab^	90.4 ± 14.9
BMI (kg/m^2^)	28.0 ± 3.7^a^	30.5 ± 4.8^b^	30.0 ± 3.9^b^	28.8 ± 4.1
Fat-free mass (kg)	63.6 ± 7.6^a^	66.8 ± 9.1^b^	59.8 ± 6.8^c^	63.6 ± 8.0
Lean mass (kg)	60.4 ± 7.3^a^	63.6 ± 8.8^b^	56.6 ± 6.5^c^	60.4 ± 7.7
Fat-mass (kg)	24.7 ± 8.6^a^	30.3 ± 10.7^b^	30.9 ± 8.4^b^	26.7 ± 9.4
BF%	27.3 ± 6.4^a^	30.4 ± 6.3^b^	33.6 ± 5.2^c^	28.9 ± 6.6
Android BF%	33.8 ± 10.5^a^	40.4 ± 9.9^b^	43.6 ± 7.0^b^	36.6 ± 10.7
Gynoid BF%	28.3 ± 6.2^a^	28.5 ± 5.9^a^	32.2 ± 5.7^b^	28.9 ± 6.2
Android/Gynoid ratio	0.56 ± 0.16^a^	0.79 ± 0.19^b^	0.81 ± 0.16^b^	0.64 ± 0.20

Values within each measure not sharing similar superscripts are significantly different, *P* < 0.01.

The mean BF% for each BMI category is displayed in **Table 3**. For all age groups, the mean BF% for the overweight category exceeded the BF% standard cut-off of ≥ 25%, i.e., 27%, 28%, and 32% for the 20–39, 40–59, and ≥ 60 year old groups, respectively.

**Table 3:** Mean BF% according to BMI classification. Values are mean ± SD with the percentage of each age group in parentheses. Body composition classification: underweight (< 18.5 kg/m^2^); normal weight (18.5–24.9 kg/m^2^); overweight (25.0–29.9 kg/m^2^); obese (≥ 30.0 kg/m^2^).

		BMI classification			
Age group	*n*	Underweight	Normal weight	Overweight	Obese
20–39 y	297	-^a^ (0.0)	21.9 ± 2.7 (23.6)	26.6 ± 5 (45.5)	32.6 ± 4.4 (31.0)
40–59 y	88	12.8^b^ (1.1)	23.7 ± 4.6 (13.6)	27.9 ± 4 (34.1)	34.2 ± 4.8 (51.1)
≥ 60 y	71	-^a^ (0.0)	25.8 ± 3.6 (5.6)	31.9 ± 4 (52.1)	36.8 ± 4.0 (42.3)
Total	456	12.8^b^ (0.2)	22.3 ± 4.7 (18.9)	27.8 ± 5 (44.3)	33.8 ± 4.7 (36.6)

^a^ *n* = 0, mean and SD could not be calculated

^b^ *n* = 1, SD could not be calculated.

For all age groups lean mass, fat mass, and BF% showed good correlation with BMI (*P* < 0.0001) (**Table 4**). BF% and BMI were significantly more correlated than lean mass and BMI and fat mass and BMI (*P* < 0.05) for individual age groups and the total group (*P* < 0.05).

**Table 4:** Pearson correlations of lean mass, fat mass, BF%, and BMI by age group.

Age group	*n*	Lean mass and BMI	Fat mass and BMI	BF% and BMI
20–39 y	297	0.589* ^a^	0.696* ^a^	0.844* ^b^
40–59 y	88	0.721* ^a^	0.783* ^a^	0.907* ^b^
≥ 60 y	71	0.573* ^a^	0.707* ^a^	0.868* ^b^
Total	456	0.589* ^a^	0.723* ^b^	0.874* ^c^

*Significantly correlated, *P* < 0.0001. Pearson correlation coefficients within each age group not sharing similar superscripts are significantly different, *P* < 0.05.

**Fig 1:**
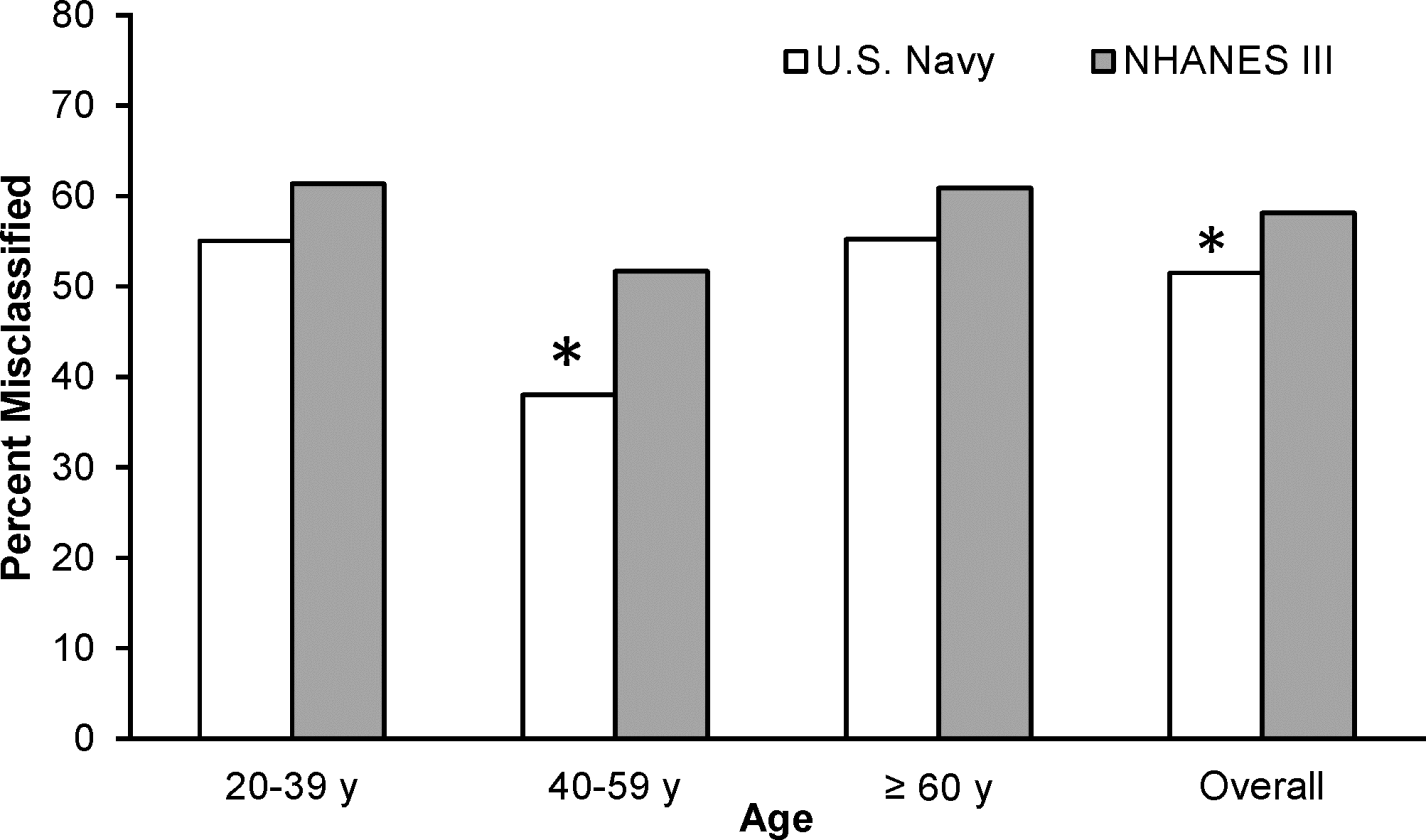
Percent of exact agreement between obesity defined by a BF% ≥ 25.0 and BMI. The percent of exact agreement peaked at BMI’s of 25 and 26 kg/m^2^.

The prevalence and exact agreement of obesity classified by BMI and BF% is shown in **Table 5**. The percent agreement between BMI and BF% in classifying obesity was fair for all submariners except those ≥ 60 years old (*P* < 0.001). The prevalence of BMI defined obesity was 37% overall, and 31%, 51% and 42% for the 20–39, 40–59 and ≥ 60 year old groups, respectively. In contrast, by using BF% to classify obesity the prevalence rates were 67% (20–39 year olds), 81% (40–59 year olds), 94% (≥ 60 year olds), and 74% overall. By using a BF% cut-off of ≥ 25%, 38% of the submariners sampled were misclassified when obesity was defined by a BMI of ≥ 30. Based on this data, we determined that a BMI cutoff of 25–26 yields the highest agreement with obesity classified by a BF% ≥ 25.0 (**Fig 1**).

**Table 5:** Prevalence and exact agreement of obesity defined by BMI and BF%. Obesity: BMI ≥ 30.0; measured BF% ≥ 25.0. Ck, Cohen’s kappa coefficient.

			Measured BF%				
Age group	*n*	BMI	Not obese	Obese	Total	Ck	Percent agreement
20–39 y	297	Not obese	32.3	36.7	69.0		
		Obese	1.0	30.0	31.0		
		Total	33.3	66.7	100.0	0.33^a^	62.3
40–59 y	88	Not obese	18.2	30.7	48.9		
		Obese	1.1	50.0	51.1		
		Total	19.3	80.7	100.0	0.36^a^	68.2
≥ 60 y	71	Not obese	5.6	52.1	57.7		
		Obese	0.0	42.3	42.3		
		Total	5.6	94.4	100.0	0.08	47.9
Total	456	Not obese	25.4	37.9	63.4		
		Obese	0.9	35.7	36.6		
		Total	26.3	73.7	100.0	0.31^a^	61.2

^a^Agreement between measured BF% and BMI significant, *P* < 0.001.

## Discussion

The prevalence of overweight and obesity within the U.S. military and veterans is similar to the general population [6, 14–16]. Although these estimated prevalence rates are from BMI data, they do provide insight into the overall severity of the epidemic. There is, however, reason to question the utility of BMI in classifying overweight and obesity in military members as the proportion of total mass that is comprised of fat-free mass may be greater as a result of physical fitness standards. Thus, determining fat and fat-free mass is required for assessing the accuracy of BMI to correctly classify military personnel as overweight and obese. From this research, there are two main findings: First, the mean BF% determined by DXA indicates that the group sampled has excess adipose tissue, similar to the general population [10]. Secondly, although there is good correlation between body composition and BMI, 38% of the subjects were misclassified as not obese by BMI when using a BF% cut-off of ≥ 25%. These data suggest that the diagnostic inaccuracy associated with BMI in obesity classification reported by others [11, 12, 25] exists within the military.

The percentage of individuals that would fall into an overweight or obese classification with BMI because of increased fat-free mass is likely small, yet may be considerable in a physically active population such as the military. In the current study, the mean BMI (28.8) and BF% (28.9) of the subjects sampled were comparable to NHANES 1999–2000, i.e., mean BMI was 27.9 and BF% determined by DXA was 28.1 [10]. If the active duty subjects are considered separately, the results are similar, i.e., mean BMI 28.2 ± 3.7 and mean BF% 27.8 ± 6.2. Of the total sample, 81% were classified as overweight (44%) or obese (37%) by BMI and 74% as obese by BF%. These data suggest that the increase in body mass is due to an increase in fat mass vs. fat-free mass, and the current estimates of overweight and obesity in active duty military members and veterans are likely not overestimated prevalence rates. Additional research with a larger sample from various geographical locations is required to confirm these findings.

There was good correlation between lean mass, fat mass, BF% and BMI, consistent with others who compared BF% and/or fat mass and lean mass with BMI in adult males [10, 11, 23, 26–28]. In the present study, BF% displayed the strongest correlation with BMI compared to lean and fat mass. This is in contrast to previous investigations that reported a stronger correlation for fat mass and BMI in adult males when measurements were made by DXA [27, 28], or quantified by a four-compartment model [26]. The weakest correlation observed in the present study was between lean mass and BMI, also observed by Gallagher et al. [26], but in contrast to Romero-Corral et al. [11]. Despite the disparity between studies as to which body mass compartment yields the strongest correlation with BMI, the most common observation is that there is a strong linear relationship between fat, either absolute or percentage, and BMI.

**Fig 2:**
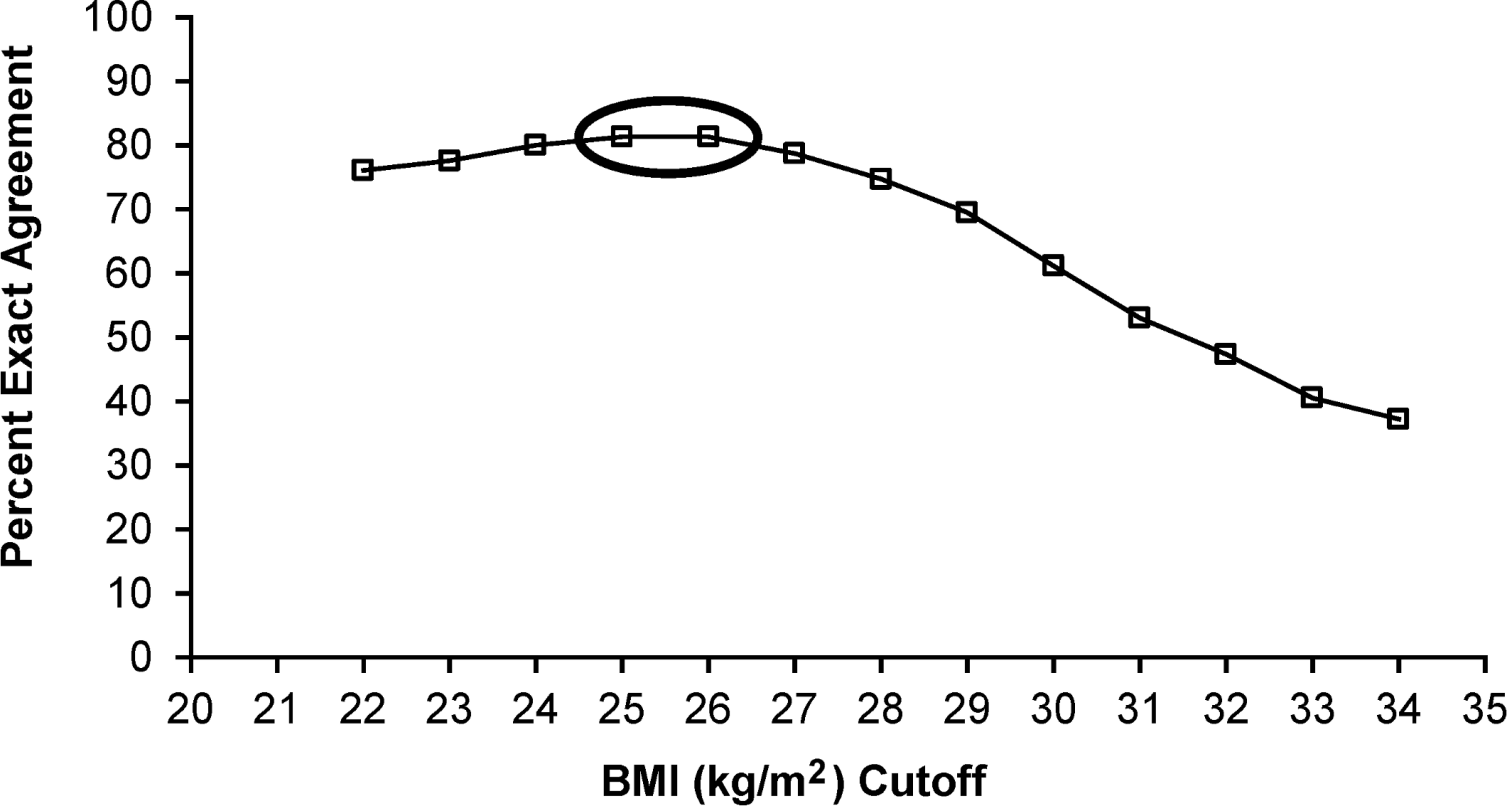
Comparison of percent misclassified as not obese for U.S. Navy and NHANES III stratified by age. Applying the BMI cutoff of ≥ 30.0, chi-square tests between samples by age group showed proportionately fewer Navy volunteers were misclassified in the 40–59 year old group and for all age groups combined (*P* < 0.05). For Navy (*n* = 336) BF% was calculated from DXA measured compartments, and for NHANES III (*n* = 3243) bioelectrical impedance was used to estimate BF% [11].

In addition to misclassifying individuals as obese when they are not, BMI may also falsely diagnose individuals as not being obese when they truly are. The classic example is an individual who appears to be at or slightly above a normal/ideal body weight, but has a larger proportion of fat mass. In the current study, BMI correctly identified 97% of the subjects that were not obese (good specificity), but only correctly identified 36% as obese (poor sensitivity); thus ~ half of the obese subjects were misclassified as being normal or overweight. The poor sensitivity of BMI to identify BF%-classified obesity is, however, well documented when BF% is determined by bioelectrical impedance [11, 23] or DXA [10, 12, 25, 27]. In our sample, the percent misclassified as not being obese by BMI was somewhat comparable to NHANES III (**Fig 2**) [11], confirming BMI is underestimating the magnitude of the obesity epidemic. If the goal is to accurately identify those who are obese, then redefining obesity cutoffs should be considered, which in our population would be 25–26 kg/m^2^.

Although the data from this research confirm a similar trend in obesity between the subjects studied and the general population, there is considerable differences in the prevalence rates for obesity related diseases, such as hypertension, hyperlipidemia and type II diabetes mellitus. Specifically, the percentage of subjects that reported being diagnosed with hypertension and type II diabetes mellitus, and hyperlipidemia, was approximately one-half and two-thirds less, respectively, than reported for similarly aged U.S. males [29, 30]. The reasoning for this difference may be due to the benefit of physical activity as the majority of the subjects, regardless of age, were obtaining sufficient daily physical activity (~150 min of moderate-intensity physical activity weekly [18]), whereas most of general population are not (~75%) [30]. While physical activity reduces an individual’s risk for developing cardiovascular disease and type II diabetes mellitus [31], these results should not discount the potential consequences of chronic obesity. Importantly, obesity was not an epidemic within the military several decades ago [32], so it is not inconceivable that the prevalence of obesity related diseases will increase over time. In order to prevent a further increase in obesity, compliance to the healthy dietary guidelines [21], which was poor overall in the subjects studied and in the general population, and increasing the duration of moderate-intensity physical activity to ~60-min most days of the week [31] is likely required.

There are limitations with this research that may affect the interpretation of the data presented herein that should be considered. First, data on a convenience sample of past and present U.S. Navy servicemembers were collected, so it is unknown as to whether these results can be generalized to the entire Navy or military. The trends in obesity defined by BMI has more than doubled between 1995 and 2008 for all military personnel 20 years or older [32]. While this does not confirm that our findings would be similarly observed in the other military branches, they do suggest that the prevalence would be significantly higher if BF% was measured by DXA, or another objective method as a result of the poor sensitivity of BMI. Second, as a result of more than 90% of the sample being non-Hispanic white, no race-ethnicity comparisons were feasible. In the U.S. Navy, ~31% of the active duty force are minorities, defined as black or African American, Asian, American Indian or Alaska Native, Native Hawaiian or Other Pacific Islander, multi-racial, and other/unknown [33]. Since the prevalence of obesity is higher in non-Hispanic black and Hispanic populations [34], this study may in fact be underestimating the problem of obesity in Navy personnel. Finally, this was a cross-sectional design and cannot determine the cause of obesity.

In summary, our results suggest that a BMI classification of overweight or obesity in a group of current and former U.S. Navy servicemembers is not due to increased fat-free mass, but adiposity. In addition, while there is a good correlation between lean mass, fat mass and BF% with BMI, and fair agreement between BF% and BMI in diagnosing obesity, a large proportion of individuals are misclassified as not being obese when using a BMI ≥ 30 cutoff as opposed to a BF% cutoff of ≥ 25%.

### Disclaimer

“The views expressed in this article are those of the author and do not necessarily reflect the official policy or position of the Department of the Navy, Department of Defense, nor the U.S. Government.”

### Copyright Statement

“I am a military service member (or employee of the U.S. Government). This work was prepared as part of my official duties. Title 17 U.S.C. §105 provides that ‘Copyright protection under this title is not available for any work of the United States Government.’ Title 17 U.S.C. §101 defines a U.S. Government work as a work prepared by a military service member or employee of the U.S. Government as part of that person’s official duties.”
